# Exostosis of Ulna With Developmental Deformity of the Left Forearm: A Rare Case

**DOI:** 10.7759/cureus.50528

**Published:** 2023-12-14

**Authors:** Sachin Goel, Nareshkumar Dhaniwala, Rahul Singh, Anmol Suneja, Vivek H Jadawala

**Affiliations:** 1 Orthopaedics, Jawaharlal Nehru Medical College, Datta Meghe Institute of Higher Education and Research, Wardha, IND

**Keywords:** benign bone tumours, multiplt heredietary exostosis, ulna, osteochondroma, exostosis

## Abstract

This case report presents a rare occurrence of exostosis of the ulna associated with a developmental deformity of the left forearm in a 15-year-old female. The patient reported a history of trauma resulting in a supracondylar humerus fracture managed conservatively eight years prior. The patient presented with a two-year history of pain and swelling over the left forearm. Clinical examination revealed a firm, non-tender, immobile swelling closely associated with the ulna, accompanied by a 20-degree cubitus varus deformity and forearm shortening. Radiographs and computed tomography scans confirmed the presence of a solitary external bony protuberance over the ulna shaft, communicating with the medullary cavity. A preliminary diagnosis of osteochondroma was established based on clinical and imaging findings. The patient underwent extraperiosteal en bloc resection of the lesion under supraclavicular nerve block anesthesia. A histopathological examination confirmed the diagnosis. Postoperative physiotherapy was initiated, and at the one-month follow-up, the patient reported being pain-free. This case highlights the rarity of exostosis of the ulna with associated developmental deformity, emphasizing the importance of a comprehensive diagnostic approach. Early surgical intervention resulted in a successful outcome, underscoring the significance of timely management in improving patient outcomes and quality of life.

## Introduction

Osteochondromas are the most common type of benign bone tumor, accounting for 35% to 40% of all benign bone tumors [1]. However, their occurrence in nonstandard locations and association with developmental deformities present unique challenges in diagnosis and management [1]. This report details an uncommon case of ulnar exostosis with a concurrent developmental deformity of the left forearm in a 15-year-old female patient, highlighting the significance of recognizing atypical presentations of osteochondromas, particularly in pediatric orthopedics.

The etiology of osteochondromas is multifactorial, often involving genetic predisposition and skeletal trauma, especially in growth plates and metaphyseal regions [2]. While these tumors primarily affect the metaphyseal regions, their manifestation in the ulna with an associated developmental deformity is rare. A comprehensive understanding of osteochondromas' pathogenesis and clinical presentations is essential for accurate diagnosis and effective management [3].

Radiographic and computed tomography (CT) imaging plays a pivotal role in characterizing the extent and nature of osteochondromas. Imaging findings offer valuable insights into these lesions' location, size, and potential complications [4]. The correlation between clinical presentation and imaging findings guides the selection of appropriate surgical interventions. Surgical excision is often necessary in symptomatic cases, with extraperiosteal en bloc resection considered the preferred approach to mitigate the risk of recurrence [5]. The surgical outcome is influenced by various factors, including the lesion's location, involvement of adjacent structures, and the patient's overall health [6].

This case report aims to contribute to understanding osteochondromas by presenting a rare case involving a unique combination of ulnar exostosis and developmental forearm deformity. By exploring the patient's clinical history, radiographic findings, and surgical intervention, we aim to enhance our understanding of the diagnostic and therapeutic challenges associated with osteochondromas in uncommon anatomical locations.

## Case presentation

**Figure 1:**
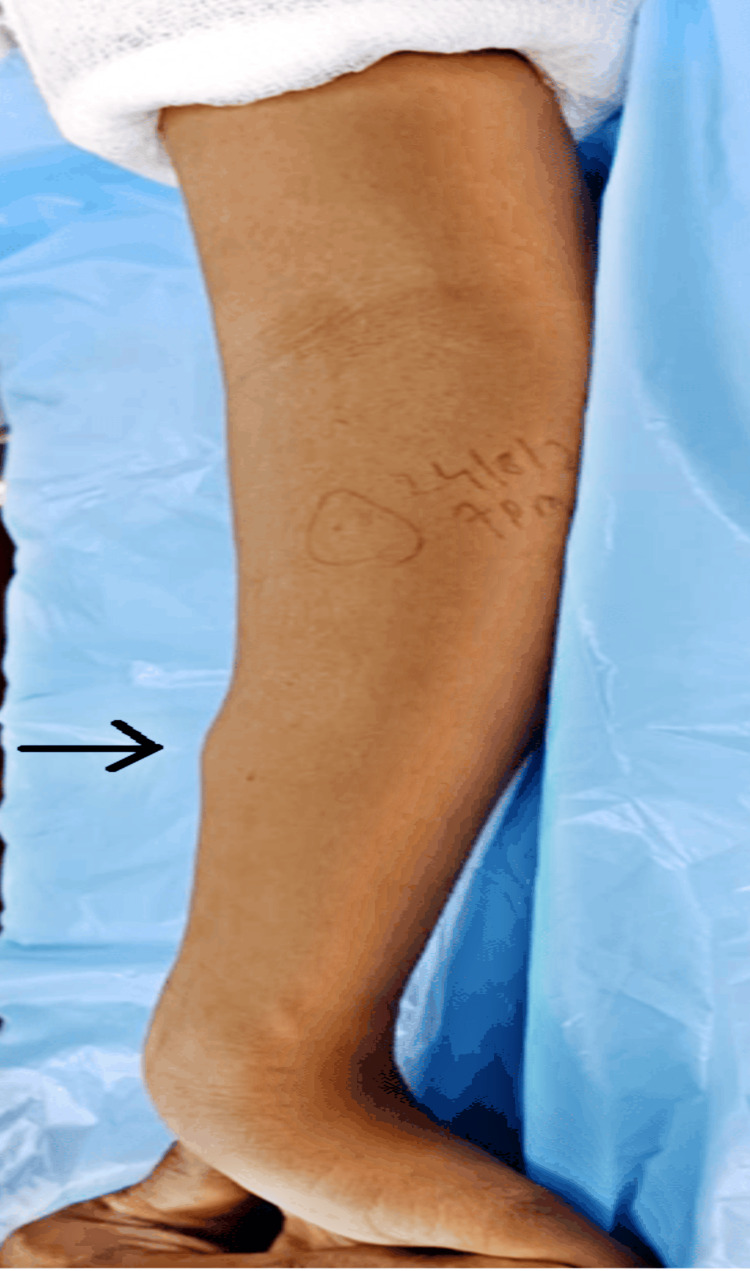
Swelling size 3 cm x 2 cm over the anteromedial aspect of the ulna

A 15-year-old female sought evaluation at the orthopedic outpatient clinic, reporting a two-year duration of pain and swelling in her left forearm. The patient recounted a traumatic incident from eight years prior, resulting in a supracondylar humerus fracture. The fracture had been treated with an above-elbow cast for three weeks. Upon examination, a firm, non-fluctuant, non-tender, and immobile swelling measuring 3 cm x 2 cm x 2 cm was identified (Figure 1). The swelling demonstrated a close association with the underlying bone and presented with normal overlying skin, showing no signs of neurovascular compromise.

**Figure 2:**
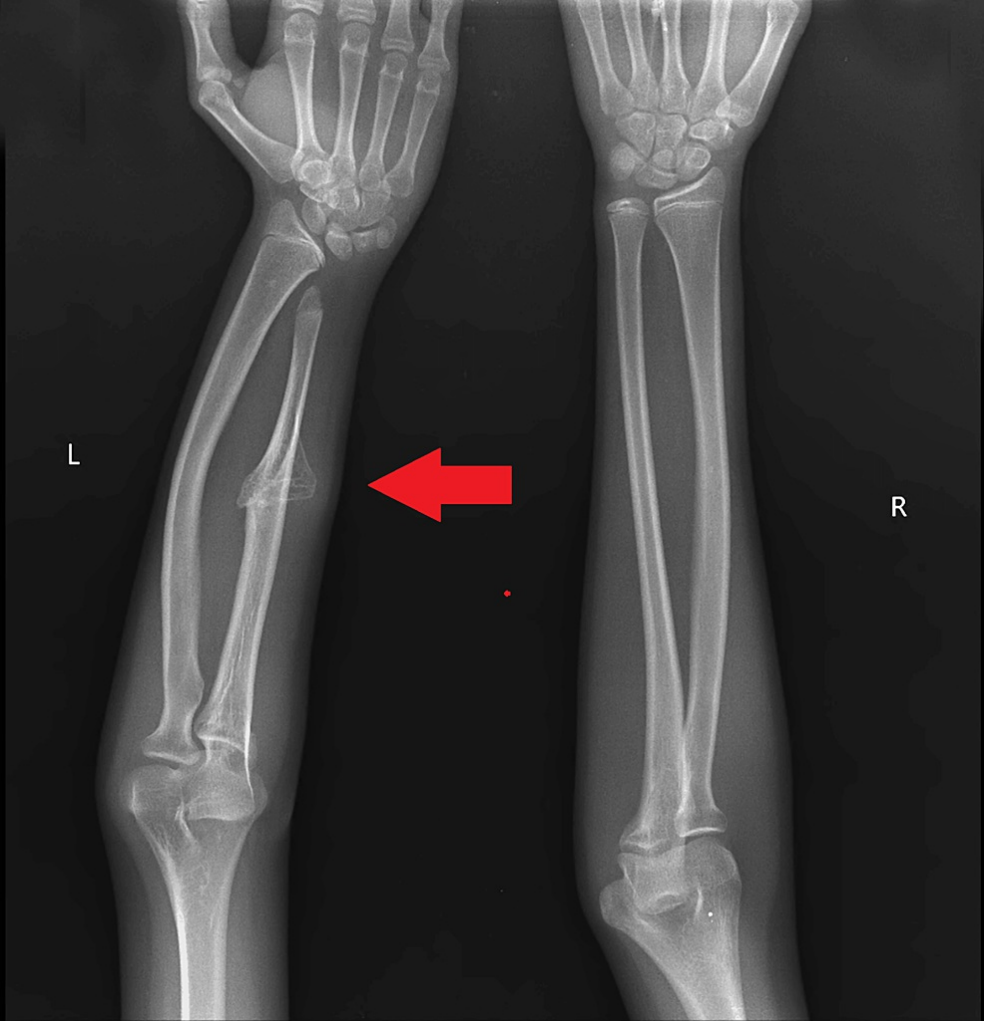
Antero-posterior X-ray of bilateral forearm showing bony protuberance over ulna and bowing of left radius

**Figure 3:**
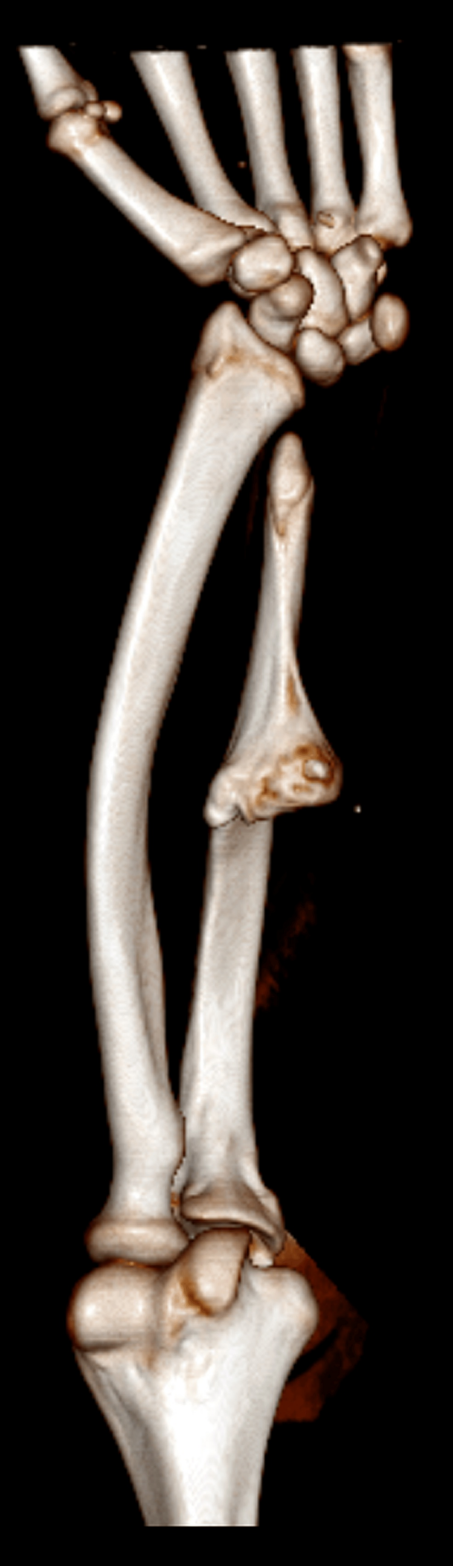
CT scan of left forearm showing large anteromedial bony projection involving the middle 1/3rd of the ulna with medullary cavity communication with the parent bone

The left elbow displayed a 20-degree cubitus varus deformity, accompanied by noticeable shortening of both the radius and ulna in the left forearm. Radiographs, encompassing anterior-posterior and lateral views, unveiled a distinct external bony protuberance over the shaft of the ulna (Figure 2). Notably, the radial styloid was absent, and the radius shaft was bowing. CT imaging provided further clarity, revealing an isolated bony protuberance on the proximal ulna. This protuberance communicated with the medullary cavity without involving the surrounding soft tissue (Figure 3).

**Figure 4:**
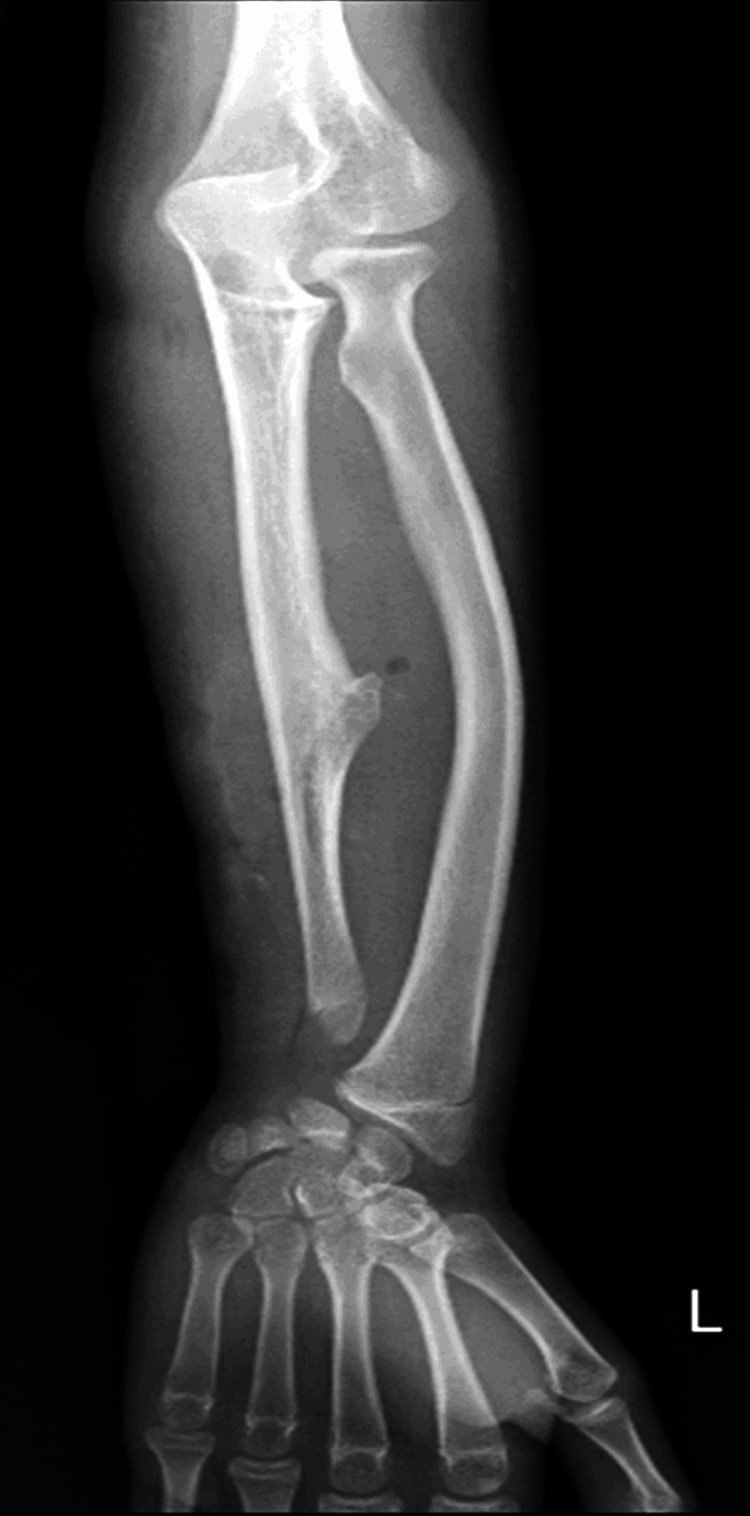
Immediate post-operative X-ray

**Figure 5:**
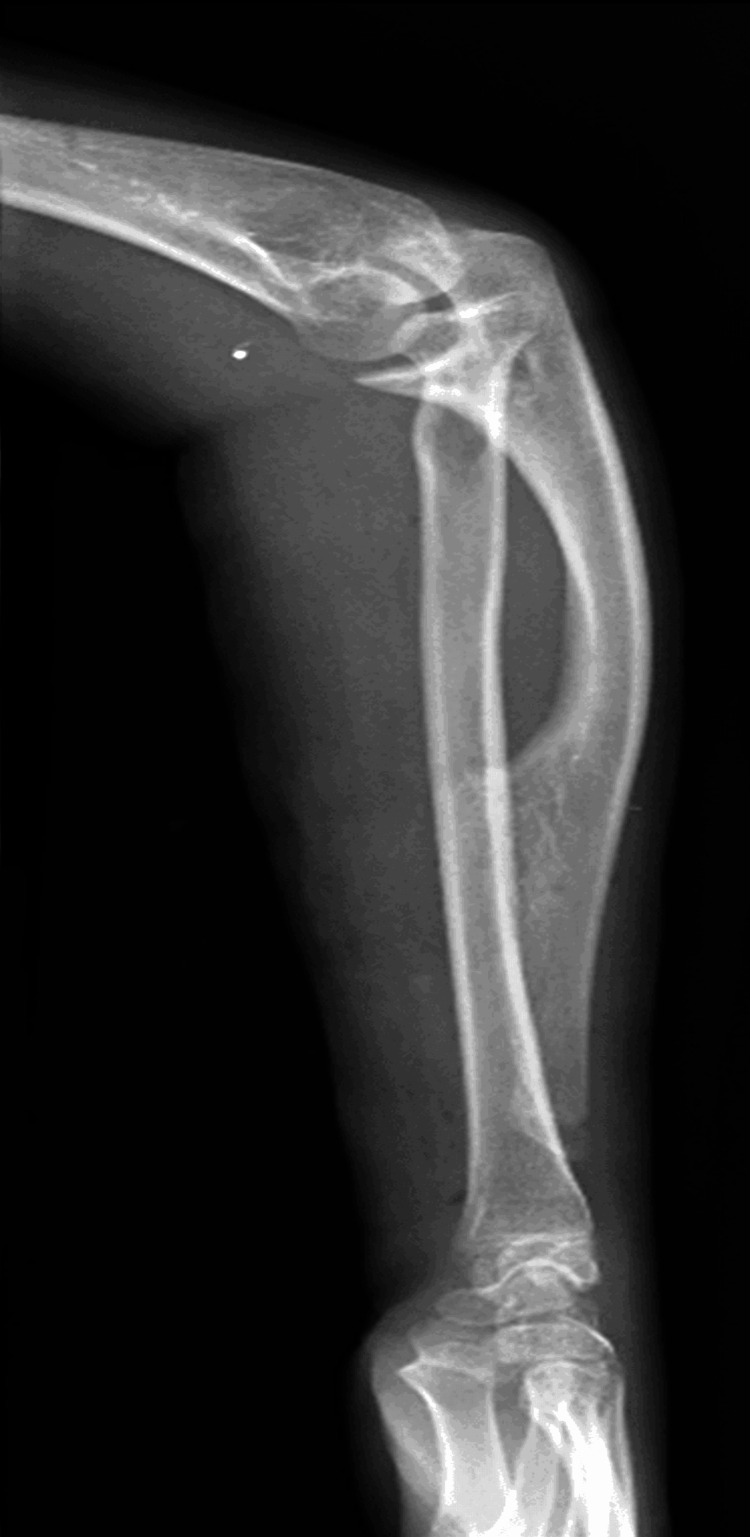
Immediate post-operative X-ray

A preliminary diagnosis of osteochondroma was established following clinical observations and imaging studies. The laboratory value of the bone is described in Table 1. Hematological investigations yielded results within normal limits, and informed consent was obtained from the patient. Subsequently, the patient underwent extraperiosteal en bloc resection of the lesion, performed under supraclavicular nerve block anesthesia. Histopathological examination of the excised specimen conclusively confirmed the diagnosis as osteochondroma. Postoperatively, radiological X-rays were obtained (Figures 4, 5). Initiation of postoperative physiotherapy exercises occurred on the first day, and suture removal was carried out on the 12th day.

**Table 1:** Laboratory value of the bone ALP: Alkaline phosphatase

Variable	Patient value	Normal range
Vitamin D	25	20 and 40 ng/mL
Parathyroid hormone (PTH)	47	10 to 55 picograms per milliliter (pg/mL)
Calcium	9.2	8.5 to 10.5 mg/dl
ALP	107	44 to 147 international units per liter (IU/L)

During the one-month follow-up, the patient reported being free of pain. This case underscores the rarity of ulnar exostosis with a concurrent developmental deformity of the forearm and highlights the importance of timely diagnosis and appropriate management for achieving optimal outcomes. Forearm deformities occur in 30% of patients with hereditary multiple exostoses, leading to radial head dislocation and loss of movement [1]. The early surgical intervention depicted in this case not only alleviated symptoms but also improved the overall quality of life for the patient.

## Discussion

The presented case of ulnar exostosis with a developmental forearm deformity in a 15-year-old female patient raises several important considerations in the field of pediatric orthopedics. The occurrence of exostosis in the ulna, combined with a history of trauma and a subsequent supracondylar humerus fracture, introduces complexity to the understanding of musculoskeletal anomalies in this population.

The patient's history of trauma and fracture aligns with previous studies that associate the development of osteochondromas with skeletal trauma, particularly in the context of growth plates and metaphyseal regions [7]. Although osteochondromas, as the most common benign bone tumors, typically originate in the metaphyseal regions, their manifestation in the ulna with a concurrent developmental deformity is relatively uncommon [8]. The specific correlation between trauma, fracture, and the subsequent development of an exostosis in this case warrants further exploration of the potential causal relationship.

Radiographic and CT imaging played a pivotal role in the diagnostic process. The anteroposterior and lateral views of the left forearm revealed a solitary external bony protuberance over the ulna shaft, providing crucial visual evidence of the pathology. CT scans further elucidated the nature and extent of the lesion, demonstrating a large anteromedial bony projection involving the middle 1/3rd of the ulna with medullary cavity communication with the parent bone. These findings are consistent with the diagnostic utility of imaging modalities in characterizing osteochondromas [9].

The surgical approach involved extraperiosteal en bloc resection of the lesion under supraclavicular nerve block anesthesia. The success of this procedure is supported by existing literature advocating for surgical excision in symptomatic cases, emphasizing the significance of addressing the lesion extraperiosteally to prevent recurrence [5]. Histopathological examination confirmed the diagnosis of osteochondroma. This aligns with previous studies highlighting these tumors' benign nature, characterized by mature hyaline cartilage capped by a bony cortex [10]. The patient's favorable postoperative outcome, evidenced by the absence of pain at the one-month follow-up, underscores the efficacy of the surgical intervention in alleviating symptoms associated with such musculoskeletal anomalies.

## Conclusions

This case highlights the significance of considering unusual etiologies, such as exostoses, in the differential diagnosis of forearm swellings, particularly in the context of a relevant medical history. The radiographic and CT findings were crucial in establishing a precise diagnosis and guiding the appropriate surgical approach. The absence of complications postoperatively and the restoration of the patient's quality of life further underscore the importance of early recognition and intervention in managing such rare musculoskeletal conditions. As our understanding of atypical presentations of common pathologies continues to evolve, this case contributes to the expanding knowledge base in orthopedics. It emphasizes the need for a multidisciplinary approach, including clinical, radiological, and histopathological assessments, to ensure accurate diagnosis and effective management. Continued documentation and sharing of such cases contribute to the collective knowledge that informs clinical practice and enhances patient care in orthopedic medicine.
